# Greening of a boreal rich fen driven by CO_2_ fertilisation

**DOI:** 10.1016/j.agrformet.2024.110261

**Published:** 2024-12-15

**Authors:** Sandeep Thayamkottu, T. Luke Smallman, Jaan Pärn, Ülo Mander, Eugénie S Euskirchen, Evan S Kane

**Affiliations:** a Institute of Ecology and Earth Sciences, University of Tartu, Vanemuise Street. 46, 51003 Tartu, Estonia; b School of GeoSciences, The University of Edinburgh, Edinburgh EH9 3FF, United Kingdom; c National Centre for Earth Observation, The University of Edinburgh, Edinburgh EH9 3FF, United Kingdom; d Institute of Arctic Biology, University of Alaska Fairbanks, Fairbanks, Alaska, USA; e Department of Biology and Wildlife, University of Alaska Fairbanks, Fairbanks, Alaska, USA; f College of Forest Resources and Environmental Science, Michigan Technological University, Houghton, Michigan, USA; g Northern Research Station, USDA Forest Service, Houghton, Michigan, USA

**Keywords:** Carbon dioxide fertilisation, Carbon allocation, DALEC, Gross primary productivity, Leaf area index, Boreal peatlands

## Abstract

Boreal peatlands store vast amounts of soil organic carbon (C) owing to the imbalance between productivity and decay rates. In the recent decades, this carbon stock has been exposed to a warming climate. During the past decade alone, the Arctic has warmed by ∼ 0.75°C which is almost twice the rate of the global average. Although, a wide range of studies have assessed peatlands’ C cycling, our understanding of the factors governing source / sink dynamics of peatland C stock under a warming climate remains a critical uncertainty at site, regional, and global scales. Here our focus was on answering two key questions: (1) What drives the interannual variability of carbon dioxide (CO_2_) fluxes at the Bonanza Creek rich fen in Alaska, and (2) What are the internal carbon allocation patterns during the study years? We addressed these knowledge-gaps using an intermediate complexity terrestrial ecosystem model calibrated by a Bayesian model-data fusion framework at a weekly timestep with publicly available eddy covariance, satellite-based earth observation, and in-situ datasets for 2014 to 2020. We found that the greening trend (a relative increase of leaf area index ∼0.12 m^2^ m^-2^ by 2020) in the fen ecosystem is forced by a CO_2_ fertilisation effect which in combination resulted in increased gross primary production (GPP). Relative to 2014, GPP increased by ∼75 gC m^-2^ year^-1^ (by 2020; 95% confidence interval (CI): -41.35 gC m^-2^ year^-1^ to 213.55 gC m^-2^ year^-1^) while heterotrophic respiration stayed constant. Consistent with the observed greening, our analysis indicates that the ecosystem allocated more C to foliage (∼50%) over the structural (A carbon pool consisting of branches, stems and coarse roots; ∼30%) and fine root C pools (∼20%).

## Highlights

• During the seven-year period, the peatland exhibited an increase in plant growth.
• The greening was driven by CO_2_ fertilisation effect.
• Carbon allocation to foliage favoured over fine root and structural carbon pools.
• We characterised uncertainties with a novel model-data fusion framework.

## 1 Introduction

Northern peatlands play a significant role in the global carbon (C) cycle by storing a third of the global soil organic C pool (∼415 ± 150 PgC (Beaulne et al., 2021; Hugelius et al., 2020)), despite only covering ∼3% of the Earth's surface (Gorham, 1991; Limpens et al., 2008; Loisel et al., 2014; Yu, 2011). In comparison, a similar amount; 409 PgC is stored in the global vegetation biomass (Spawn et al., 2020). Peatlands, in their natural hypoxic state are net sinks of carbon due to lower rates of decomposition compared to C fixed by the plants (Mäkiranta et al., 2018, Sonnentag et al., 2010). These organic soils are exposed to intensified climate warming (Huang et al., 2017; Post et al., 2019) and extreme events such as droughts, leading to a water table decline (Ma et al., 2022). The Arctic has warmed at ∼4 times faster than the rate of the rest of the planet during the industrial period (IPCC, 2023, Myers-Smith et al., 2020, Post et al., 2019, Rantanen et al., 2022). These events can start a domino effect of climatic responses (Belyea, 2009). For example, the vapour pressure deficit (VPD) in the boreal biome alone has increased ∼10% since the late 2000s (Helbig et al., 2020) and resulted in increased evapotranspiration. Warming could extend the period of thaw, shorten winter, prolong the growing season, and trigger flooding during the C uptake period. Sources and timing of these floodings can have significant impact on the inter-annual variability of C (Euskirchen et al., 2020). For instance, water table drawdown could trigger enhanced decomposition or gross primary productivity (GPP), shifts in phenology and community structure (Antala et al., 2022; Davidson et al., 2021; Guay et al., 2014; Peichl et al., 2018; Wilkinson et al., 2023; Zhang et al., 2018). Satellite-based Earth Observation (EO) provided indices of vegetation cover and leaf area have shown positive trends which indicate increased photosynthetic activity and potentially an associated increases in biomass, canopy cover/leaf area (Berner et al., 2020; Guay et al., 2014; Ju and Masek, 2016; Myers-Smith et al., 2020). On the other hand, a warming induced browning effect is also reported in parts of the Arctic (Myers-Smith et al., 2020; Phoenix and Bjerke, 2016).

A multitude of controlled experiments have looked at the impact of elevated temperature, water table fluctuations, and atmospheric CO_2_ concentrations on plant growth, growing season length and C exchange (Chivers et al., 2009; Kane et al., 2021; McPartland et al., 2019; Olefeldt et al., 2017). While these studies shed light into climate change impact on peatland C cycle, it is limited to number of sites, scarce number of biotic and abiotic factors and to major fluxes such as net ecosystem exchange (NEE), ecosystem respiration (Reco), and GPP. Hence it is imperative to include peatland CO_2_ dynamics in process-based models.

Several recent studies have focused on process-based modelling of high latitude peatland C accumulation, decomposition rate and climatic influence in the past and future (Belyea and Malmer, 2004; Chaudhary et al., 2020; Gorham et al., 2012; Qiu et al., 2020; Yu, 2011; Zhang et al., 2018). However, uncertainties remain in the magnitude, extent, and inter-annual variability of climate-carbon feedback, as is the role of multiple biogeochemical processes. This includes the peatland C allocation patterns and its response to climate warming. Recent field and lab experiments found contrasting evidence for allocation patterns under increased temperature (Tian et al., 2020, Zeh et al., 2022) leading to differences in soil respiration estimates (Walker et al., 2016, Zeh et al., 2022). This can be further complicated by CO_2_ fertilisation as it is the only negative feedback (increased uptake of CO_2_ by terrestrial ecosystems). Models, field, and EO driven studies have reported varying sensitivity of the terrestrial ecosystems to increased atmospheric concentration of CO_2_ (Chen et al., 2024; Keenan et al., 2023; Tian et al., 2020; Smith et al., 2016; Liu et al., 2019). These differences could contribute significantly to the C budget especially in the Arctic and sub-Arctic ecosystems. Consequently, net C uptake, its allocation to plant tissues (e.g. foliar, litter, and root), their residence time, and C stock of live and dead biomass pools under changing climate remain especially poorly understood.

In light of these knowledge gaps, we investigated the boreal peatland plant C cycling under the warming climate using an intermediate complexity model; Data Assmilation Linked Ecosystem Carbon model version 2 (DALEC2) (Bloom and Williams, 2015; Williams et al., 2005) calibrated using a Bayesian model-data fusion (MDF) framework called CARbon DAta MOdel fraMework (CARDAMOM) (Bloom et al., 2016). CARDAMOM probabilistically estimates the parameters of DALEC2 using a combination of eddy covariance (EC) information and EO for Bonanza Creek rich fen in Alaska, USA. In this study we seek answer to two research questions and test four hypotheses (H):

1. What drives the interannual variability of CO_2_ fluxes at the site?
  a. H_1_: Production has increased due to CO_2_ fertilisation
  b. H_2_: A reduction in decomposition has driven the C balance.
2. What is the internal carbon allocation patterns during the study years?
  a. H_3_: Fractional allocation of photosynthate will be greater to foliage than fine root
  b. H_4_: Fractional allocation of photosynthate to wood / structural C (DALEC2 representation of the structural C includes coarse roots) will be significantly greater than foliage.

## 2 Materials and methods

In this study, a Bayesian MDF framework, CARDAMOM, is used to calibrate an intermediate complexity model of the terrestrial ecosystem (DALEC2) at site scale using location specific biophysical and biogeochemical observations (outlined below). We examined the meteorological and biophysical factors that govern the interannual variability of CO_2_ fluxes and plant C traits describing internal dynamics such as photosynthate allocation to different plant tissues, residence time of C in the live and dead biomass pools by considering a seven-year period at weekly time-step (2014-2020). For this, we initialized the model with in-situ observations of soil organic carbon stock (SOC), aboveground biomass (AGB), and fine root C stock (Table 1). The model was calibrated with publicly available EC tower dataset (US-BZF, (Euskirchen, 2022a)), and EO. We used the model retrieved C variables (NEE, GPP, Reco, and LAI) to investigate the reasons behind the inter-annual variability of plant C dynamics and C uptake at the site scale. Additional synthetic experiments on the calibrated model were caried out to examine the individual effects of the climatic drivers on the inter-annual variability of GPP, LAI and heterotrophic respiration (R_h_). The study site, model inputs, model, Bayesian framework, synthetic experiments, and statistical tests performed are outlined below.

**Table 1:** *In-situ* measurements used for the model calibration and validation with CARDAMOM.

Parameter	Value ± SE	Unit	Relevant literature
Total NPP	214.33 ± 150.5	gC m^-2^ year^-1^	Churchill et al. (2015)
Belowground (BG) NPP	34.8 ± 10.165	gC m^-2^ year^-1^	Churchill et al. (2015)
Aboveground (AG) NPP	186.5 ± 63.16	gC m^-2^ year^-1^	Churchill et al. (2015)
Aboveground carbon stock*	282 ± 49.165*	gC m^-2^	Churchill et al. (2015)
Fine root carbon stock*	247.06 ± 140.86	gC m^-2^	McConnell et al. (2013)
Soil organic carbon (SOC) stock*	64055 ± 5000	gC m^-2^	Fan et al. (2013)

*Assimilated data.

### 2.1 Study site

Bonanza Creek is a rich fen peatland situated ∼45 km southeast of Fairbanks in interior Alaska (64.82°N, 147.87 W). The site is positioned within the Tanana River floodplain and is characterised as rich fen (pH 5.2-5.4). The vegetation is comprised of marsh cinquefoil (*Potentilla palustris*), wheat sedge (*Carex atherodes*), water horsetail (*Equisetum fluviatile*) and a ground cover mostly comprised of brown mosses (*Drepanocladus aduncus* and *Hamatocaulis vernicosus*) and sparse *Sphagnum* spp. (Euskirchen, 2022). The site is part of a long-term ecological experimental study dating back to 2005. The total above ground carbon stock for the year 2009 was 282 (SE: ± 49.165) gC m^-2^ and the ancillary biomass data indicates an above ground net primary productivity (NPP) of 186.5 (± 63.16) gC m^-2^ year^-1^ and a maximum vascular green area of around 2.5 m^2^ m^-2^, achieved in summer season. The depth of peat is ∼1 m and is within an area of discontinuous permafrost with seasonal freezing. The long-term (1917-2000) mean annual temperature is -3.1°C. In comparison, the mean annual air temperature for 2014-2020 was -0.95°C. In accordance with the updated Köppen-Geiger climate classification scheme, the site falls within the sub-Arctic climate (continental, warm summer, and without a dry season; Dfc zone) (Kottek et al., 2006).

### 2.2 Inputs

We used an array of observations spanning in-situ inventory and EC, along with EO and databased information. NEE and night-time partitioned Reco (Reichstein et al., 2005) were extracted from EC (US-BZF, Euskirchen, 2022a). We chose night-time partition modelled data over day-time partition because it aligns with the in-situ GPP (Churchill 2011; Fan et al., 2013; Churchill et al., 2015). Uncertainty associated with NEE was assumed to be 0.58 gC m^-2^ day^-1^ based on an analysis by Hill et al. (2012) and previously applied in CARDAMOM (e.g. Famiglietti et al., 2021). Reco uncertainty is composed of the NEE uncertainty plus the mean mass balance mismatch from the flux partitioning (0.16 gC m^-2^ day^-1^) and inflated to account for uncertainty in the partitioning approach totalling an uncertainty of 1 gC m^-2^ day^-1^. Time series information on LAI was extracted from the 300m Copernicus product (Fuster et al., 2020), using the product provided uncertainty but with a minimum bound of 0.5 m^2^ m^-2^ to account for model-structural uncertainty. Continuous LAI time series were only available for the months starting from April until September for most of the seven-year period, while the rest were absent because of the deciduous nature of the plant foliage. We assimilated NEE, LAI, and Reco at weekly timestep and the in-situ SOC stock and fine root C stock were used for the model initialisation. The aboveground C stock data was assimilated as time series where it was used as a point in the first week of 2014, leaving the rest of the time steps empty. We assimilated EC and LAI data from the alternating years (2014, 2016, 2018, 2020). The remaining EC and in-situ data were used for model validation (see Table 1 for the list of *in-situ* data).

### 2.3 Climatic drivers

A set of weekly time-step meteorological data (SI table 2) was required as model drivers. We used the atmospheric CO_2_ concentration for 2014, 2015 and the first six months of 2016 from the nearby black spruce forest EC data (US-BZS, Euskirchen, 2022b), since the measurements for this period were unavailable for the rich fen peatland.

### 2.4 DALEC2

**Fig 1:**
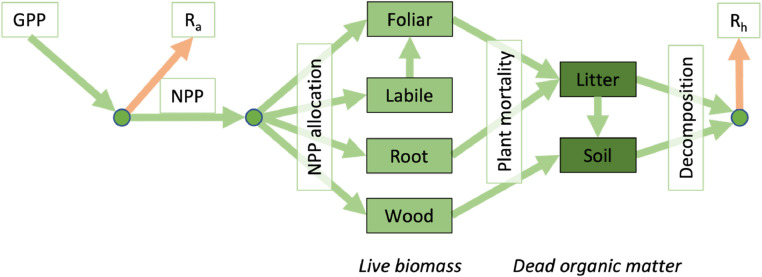
DALEC2 model carbon path.

DALEC2 is a C mass balance model representing four live biomass pools and 2 dead biomass pools (Fig. 1). Parameters within DALEC2 represent the initial C states in the first time step and define the internal C-cycling and their sensitivity to the environment (Table S1).

Photosynthesis, GPP, is estimated as a function of leaf area, canopy photosynthetic capacity, temperature, shortwave radiation, and atmospheric CO_2_ concentration by the aggregated canopy model version 1 (ACM-1) (Williams et al., 1997). Autotrophic respiration (R_a_) is estimated as a fixed parameterizable fraction of GPP, the remainder being NPP. NPP is then allocated to the four live biomass pools using fixed fractions retrieved during the calibration process. Canopy growth is determined by a combination of direct allocation of NPP and C supply from a labile pool based on a day of year model. Canopy senescence to a litter pool is determined by a day of year model. The litter pool is either decomposed to SOC or released as R_h_ based on an exponential temperature function. Mineralisation of SOC also follows an exponential temperature function.

### 2.5 CARDAMOM

The CARDAMOM MDF framework uses a Bayesian approach with an Adaptive-Proposal Markov Chain Monte Carlo (AP-MCMC) algorithm (Haario et al., 2001) to retrieve ensembles of model parameters and pool sizes (28 parameters; Table S1) for a model that are consistent with the observations, their uncertainties and ecological theory embedded in ecological and dynamical constraints (EDCs, Bloom and Williams, 2015). The use of MCMC allows uncertainty characterisation without assuming the shapes of the distribution. The MCMC with the help of EDCs, uniform prior ranges (Table S1), and initial prior estimates (where applicable) retrieve a sample parameter hyperspace. This then becomes the new prior estimate which is then is tested against the assimilated data and draws new sets of parameters that are consistent with EDCs and the other samples are rejected. CARDAMOM analyses each time step three times (known as chains) independently and assess 100 million parameter proposals in each chain. 100 subsamples (parameter ensembles) are drawn from each of the three chains for estimating posterior probability density estimates for each of the 28 parameters. The retrieved parameter ensembles allow us to directly quantify parameter uncertainty and through simulating these ensembles, the uncertainty in the ecosystem C stocks and fluxes. We normalised the likelihood estimates of the posterior parameter probability estimates generated by CARDAMOM by calculating square root to balance multiple data constraints and the imbalance between large volume of EC data compared to the field observations (Wutzler and Carvalhais, 2014).

### 2.6 Statistical analysis performed on the model posteriors

CARDAMOM calibrated weekly time-step posterior probability distributions of C variables, meteorological drivers, and time invariant parameters along with their characterised uncertainties at 95% confidence interval (CI, it is estimated using quantiles (in fractional form); 0.025, 0.5 and 0.975) were averaged at an annual scale. To assess the associations between fluxes, parameters, and climatic factors, we calculated the changes in CARDAMOM profiled probability estimates of C fluxes relative to the year 2014 to find the driving factors behind the growth in the production in the ecosystem. Further corroborative analysis using simple linear regressions answering our research questions were also made.

### 2.7 Experiments to analyse the individual contributions of the climatic drivers in the CO_2_ balance

We designed two simulation experiments on the calibrated DALEC model for extracting the information on the independent contribution of the meteorological drivers in CO_2_ exchange. To do this we repeat the retrieval of the C variables at a fixed atmospheric CO_2_ concentration (400.584 ppm, this estimate represents the atmospheric CO_2_ concentration on the first week of the study period. and simulated DALEC and retrieved the C variables. This was then compared with the original CARDAMOM calibrated DALEC estimates. These original CO_2_ estimates (from the sensor onboard the EC tower) on average were increasing during the study period (mean annual estimate is ∼ 432 ppm). In the second experiment, we removed the interannual variations in the meteorological drivers (except atmospheric CO_2_ concentration) aggregating by the weekly timesteps and estimated the mean across the seven-year study period (estimated mean of week 1 for each year, week 2 for each year and so on). We repeated the retrieval of the C variables again and compared against the CARDAMOM profiled DALEC estimates.

We then isolated the direct CO_2_ fertilisation effect and the indirect effect through the changes in LAI by driving ACM-1 with the respective model drivers of the two experiments. The difference in ACM-1 estimated GPP and the respective experiments represent this indirect effect.

All the analysis on the posterior probability estimates generated by CARDAMOM and outputs from the tests performed on the calibrated model were done using the R programming language version 4.1.3 (R Core Team, 2022) in RStudio version 2023.12.0.369 (Posit team, 2023).

## 3 Results

### 3.1 Carbon balance

**Fig 2:**
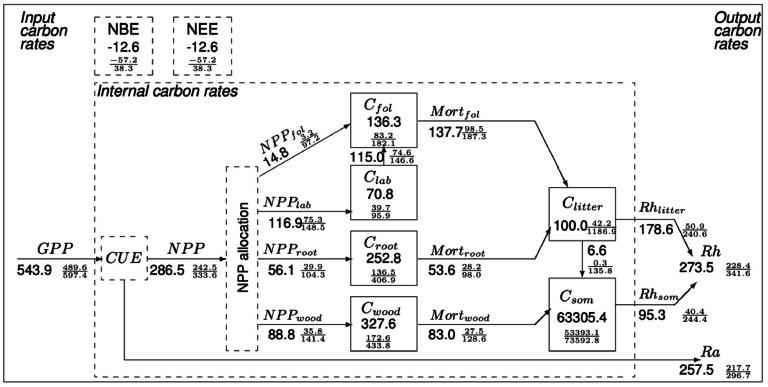
CARDAMOM C-cycle analysis of Bonanza Creek rich fen peatland (AK-US), weekly time step between 2014-2020. Numbers show median estimate of fluxes (alongside arrows) and of stocks (in boxes). Units are gC m^-2^ for stocks and gC m^-2^ year^-1^ for fluxes. 95% confidence intervals are shown in a fractional form with 2.5 and 97.5 percentiles as numerator and denominator. Carbon use efficiency (CUE) is the ratio of NPP to GPP. Black fluxes are biogenic, including NPP, mortality (Mort), R_a_, and R_h_. NEE = R_a_ + R_h_ − GPP. Net biome exchange (NBE) = NEE – Disturbance fluxes. Since there were no fire events, NBE is the same as NEE. Wood in the subscripts indicates structural C (coarse roots, stems, and branches where applicable) of the plants.

We provide a simplistic representation of the peatland plant C cycle dynamics (Fig. 2; These estimates are generated from weekly timestep estimates simulated by CARDAMOM (Fig S1). We validated the CARDAMOM estimates with the data which were not part of the assimilation (Fig S2)) starting with GPP. CARDAMOM calibrated DALEC model estimated, the fen peatland on an average fixed 543.9 gC m^-2^ year^-1^ as GPP with an uncertainty (95% CI) ranging from 489.6 gC m^-2^ year^-1^ to 597.4 gC m^-2^ year^-1^. From this, a NPP of 286.5 gC m^-2^ year^-1^ (95% CI: 242.5 g C m^-2^ year^-1^ to 333.6 gC m^-2^ year^-1^) was allocated to the four live biomass pools represented in DALEC, giving a plant carbon use efficiency (CUE, NPP: GPP) of ∼0.52. While the foliage C pool got 129.8 gC m^-2^ year^-1^ (95% CI: 77.8 gC m^-2^ year^-1^ to 243.8 gC m^-2^ year^-1^) for the seven years, the fine root and structural C pools received only 56.1 gC m^-2^ year^-1^ and 88.8 gC m^-2^ year^-1^ respectively. These estimates resemble the field data closely (Table 1, Table S3). We estimate that labile C pool takes up the second largest share of the NPP after foliage (116.9 gC m^-2^ year^-1^ with 95% CI: 75.3 gC m^-2^ year^-1^ to 148.5 gC m^-2^ year^-1^) which is then used for leaf flushing during the spring onset.

DALEC estimate of the fine root C stock; 252.8 gC m^-2^ (Fig. 2 & 5b, 95% CI: 136.5 gC m^-2^ to 406.9 gC m^-2^) accurately represent the biophysical conditions at the site (in-situ data 247 ± 145 gC m^-2^, (McConnell et al., 2013; A meta analysis of published literature is provided in Table S3). CARDAMOM profiled mean annual estimates of structural, labile and foliage C stock were 327.6 gC m^-2^ (95% CI: 172.6 gC m^-2^ to 433.8 gC m^-2^), 70.8 (95% CI: 39.7 gC m^-2^ to 95.7 gC m^-2^) and 136.3 gC m^-2^ (95% CI: 83.2 gC m^-2^ to 182.1 gC m^-2^) respectively (Figs. 1 & 5b). From this live biomass pool, due to foliage and fine root mortality, litter C stock accounts for 100 gC m^-2^ (Fig. 5b; 95% CI: 42.2 gC m^-2^ to 1186.9 gC m^-2^) annually. CARDAMOM also estimated 6.6 gC m^-2^ year^-1^ (0.3 gC m^-2^ year^-1^ to 135.8 g C m^-2^ year^-1^) to be part of the organic C stock annually from the litter C pool and 83.0 gC m^-2^ year^-1^ (27.5 gC m^-2^ year^-1^ to 128.6 gC m^-2^ year^-1^) from the structural (woody) C pool. These two dead biomass pools contributed 178.6 gC m^-2^ year^-1^ (50.9 gC m^-2^ year^-1^ to 240.6 gC m^-2^ year^-1^) and 95.3 gC m^-2^ year^-1^ (40.4 gC m^-2^ year^-1^ to 244.4 gC m^-2^ year^-1^) in that order to an annual R_h_ (273.5 gC m^-2^ year^-1^ (228.4 gC m^-2^ year^-1^ to 341.6 gC m^-2^ year^-1^)).

### 3.2 The inter-annual variability of CO_2_ fluxes

**Fig 3:**
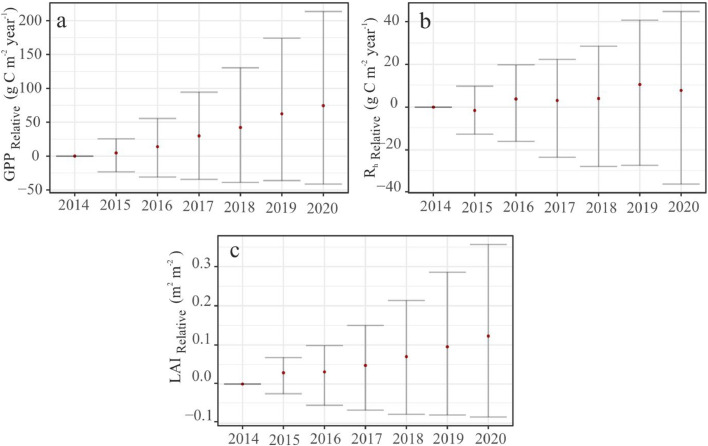
CO_2_ balance of Bonanza creek explained by relative change in C variables. a): GPP, (b): R_h_, and (c): LAI. The points indicate the relative change in median estimate with respect to 2014. The error bars represent relative change in the 95% CI from the year 2014. These are estimated based on the CARDAMOM calibrated DALEC weekly time-step outputs.

We found the increasing net carbon uptake was most likely driven primarily by a positive trend seen in the photosynthesis (Fig. 3a; and 6a (R^2^_adj_ = 0.97, p value= 2.62e-05)) and a stable R_h_ (Fig. 3b).

**Fig 4:**
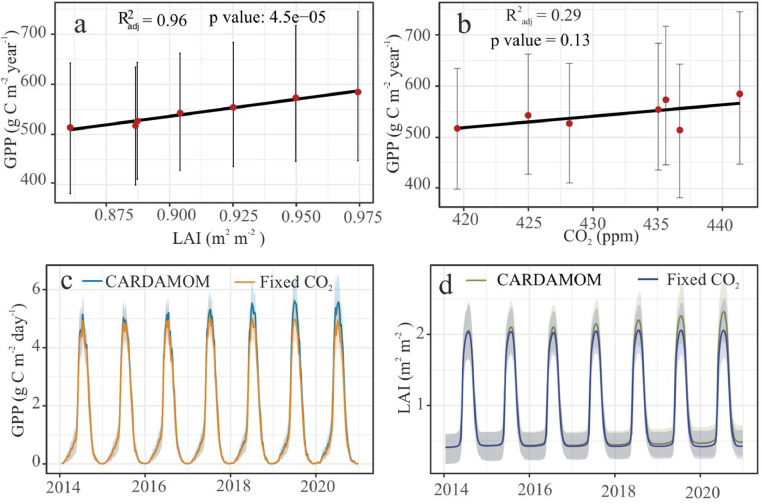
The greening and the subsequent increase in the production is forced by CO_2_ fertilisation effect. (a): The positive inter-annual trend seen in GPP is explained by the (a): LAI, and (b): atmospheric CO_2_ concentration. Comparison between the weekly posterior probability estimates of (c): GPP and (d): LAI, profiled by CARDAMOM, and the Fixed CO_2_ experiment. The shaded regions represent the respective 95% CI. For more details on the synthetic experiments, refer the methods section 2.7

This is corroborated by the increasing LAI (Figs. 3c & 4a). Relative to 2014, GPP exhibited a steady growth of around 75 gC m^-2^ year^-1^ (95% CI: -41.35 gC m^-2^ year^-1^ to 213.55 gC m^-2^ year^-1^) by 2020 (Fig. 3a). The relative increase of R_h_ by ∼8 gC m^-2^ year^-1^ (95% CI: -36.03 gC m^-2^ year^-1^ to 44.75 gC m^-2^ year^-1^) (Fig. 3b) was not enough to offset this growth in photosynthetic capacity. This could be because the rich fen peatland was flooded (∼40 cm above the peat surface) for most of the growing seasons (Euskirchen et al., 2024). We think any increase in the mean annual temperature (Fig. 6c) was not enough to cancel out this effect and the flooding pointed to an absence of SWC limitation of GPP and NEE (Fig. 7).

### 3.3 Elevated atmospheric CO_2_ concentration drives production

The increase in GPP seen above (Fig. 3a) was caused by the impact of LAI (Fig. 4a; R^2^_adj_ = 0.96, p value = 4.5e-05, residual standard error (RSE) = 5.35 with 5 degrees of freedom (DF)) and CO_2_ fertilisation effect (Fig. 4b, R^2^_adj_ = 0.29, p value = 0.13, RSE = 23.53 at 5 DF). Our synthetic experiments show that compared to the original CARDAMOM estimates (marked as CARDAMOM in Fig. 4c & 4d), the fixed CO_2_ experiment revealed a decline in GPP (Fig. 4c) and LAI (Fig. 4d). DALEC revealed similar effects in the NEE and Reco estimates (Fig. S3). The Fixed Climate experiment did not show any independent climate–carbon feedback (Fig. S4) suggesting that the effect is restricted to CO_2_ fertilisation. Furthermore, we did not see any changes in GPP compared to CARDAMOM estimates, when the sub-model ACM-1 was driven by the same synthetic meteorological datasets used in the fixed CO_2_ and climate experiments and thus separated the indirect CO_2_ fertilisation effect through changes in LAI (Fig. S5).

### 3.4 Foliage allocation is favoured over the fine root and structural C pool allocation

**Fig 5:**
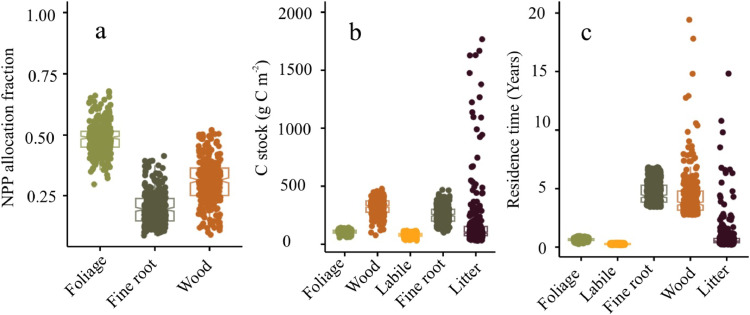
a: Mean annual NPP allocation fractions, b: C stock, and c: mean residence time of the five C pools estimated from the 300 ensemble members. The points indicate respective ensemble members.

Foliage received the largest allocation of NPP (Fig. 5a). On an average 50% of the NPP is foliage allocation, ∼30% of NPP is allocated to woody (i.e. structural, including coarse root) C pool with the remaining 20 % of NPP allocated to fine roots (Fig. 5a). Our analysis estimates that the foliage C pool has a residence time of six to eight months (Fig. 5c).

The fine roots and woody C pools are estimated to have a C residence time of 4.6 (95% CI: 4.5 to 4.7 years) years (This is supported by published literature. See Table: S3).

## 4 Discussion

Research on peatland C cycling, associated phenological and biophysical traits including carbon allocation patterns and residence times of the C pools is a necessity and seldom explored to our knowledge. We investigated the inter-annual variability of the major C fluxes (GPP, Reco), internal C allocation patterns and C residence times of a sub-arctic lowland boreal rich fen peatland using model-data fusion. We found that the increasing production is driven by CO_2_ fertilisation by designing two experiments. We provide a novel and innovative Bayesian calibration framework, CARDAMOM which can be applied for estimating the plant C cycle budget changes and its internal C traits. CARDAMOM will provide probabilistic estimates of the model parameters and its available inputs accounting for the quantity, their type, and associated errors. For this CARDAMOM uses APMCMC to run the model millions of iterations and search for parameter posteriors that are consistent with the observations. This allows CARDAMOM to produce large parameter ensembles making uncertainty characterisation possible. As opposed to traditional models, CARDAMOM does not impose strict steady state and thus avoids model spin up of 100s of years to attain steady state. Here CARDAMOM use the EDCs to reach a quasi-steady state if there is no clear parameter knowledge. This uncertainty will be registered when determining whether the ecosystem is a source of C or not (Williams, 2022).

### 4.1 Inter-annual variability of CO_2_ fluxes

**Fig 6:**
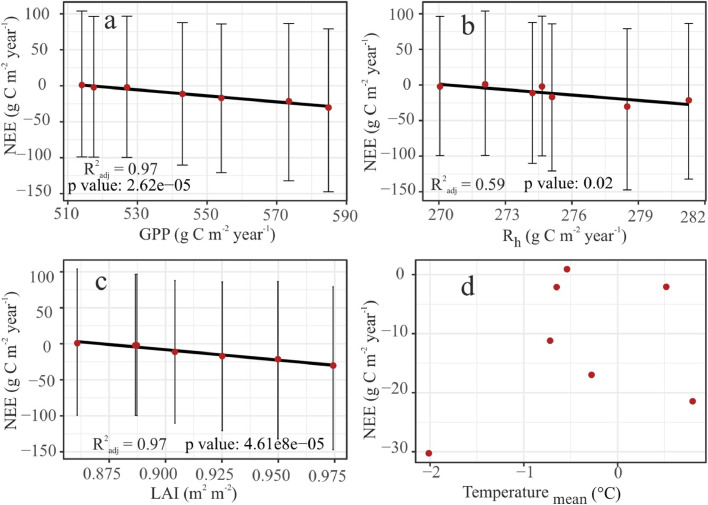
Association within CARDAMOM profiled median estimates of a: GPP & NEE, b: R_h_ & NEE, c: LAI & NEE, d: mean annual temperature and NEE (marked as red points). The error bars represent the 95% CI.

The fen peatland exhibited an increase in production and R_h_ failed to counterbalance it. Since we calibrated DALEC2 with weekly time-step EC tower and EO data for a reasonably longer time-period of seven years, the model may have captured any short-term perturbance in the climate and biases in the data inputs which is accounted for in the uncertainty characterisation. This estimation of the error propagation is a key factor for detecting trends in the inter-annual variations (Baldocchi et al., 2018). We found that the uncertainty dominated the mean estimates (Figs. 2, 6a, b, & c; RSE: 1.91, R^2^_adj_ = 0.97, F-statistic: 216.3 on 1 and 5 DF, p-value: 2.627e-05; RSE: 7.38, R^2^_adj_ = 0.59, F-statistic: 9.918 on 1 and 5 DF, p-value: 0.0254; and RSE: 2.14, R^2^_adj_ = 0.97, F-statistic: 171.7 on 1 and 5 DF, p-value: 4.618e-05 respectively). This uncertainty could originate from the lack of sufficient data on the pool sizes, internal fluxes, and model structure (Table 1, Table S1). For example, litter, and fine root C stock uncertainty have a huge 95% CI of 42.2 gC m ^-2^ to 1186.9 gC m^-2^ and 136.5 gC m^-2^ to 406.9 gC m^-2^ respectively (Figs. 2, & 5). Though, the trend in the mean annual estimates of CO_2_ fluxes implies at plant growth, we did not observe an explicit interannual variation. This is partly due to the uncertainty in the interannual estimates.

Several recent studies have shown a production-driven greening effect globally (Piao et al., 2020) and in the Arctic (Ackerman et al., 2017; Arndt et al., 2019; J. Jia et al., 2009; Myers-Smith et al., 2011, 2015, 2020). This implies that the browning trend reported by Phoenix & Bjerke (2016) for the period 2011 to 2014 may have been a local short-term temperature acclimation trend. Although the global and Arctic greening trend has been explained by land surface temperature (Hopple et al., 2020; Li et al., 2023), our results do not show temperature sensitivity of GPP. Instead, the increase in GPP is driven by higher atmospheric CO_2_ concentration.

### 4.2 CO_2_ fertilisation effect on greening

We separated the impact of atmospheric CO_2_ concentration on GPP from climatic drivers (minimum and maximum temperature, incoming shortwave radiation, and precipitation) by two synthetic experiments with the calibrated model. In the Fixed CO_2_ experiment we fixed atmospheric CO_2_ at 400.584 ppm and left the other model drivers unchanged. The experiment revealed CO_2_ concentration as the limiting factor for primary production in the rich fen (Fig. 4, Fig. S3). We also separated the effect of CO_2_ fertilisation through changing LAI.

A manipulation experiment on monoliths from a sedge-dominated boreal peatland in Canada showed the impact of temperature and Atmospheric CO_2_ (Tian et al., 2020). Our work, to the best of our knowledge, is the first long-term ecosystem-scale study to partition between effects on peatland C dynamics. While we found evidence of CO_2_ fertilisation effect on peatland production, temperature (McPartland et al., 2019) and other climatic factors including SWrad and precipitation did not show any impact on plant productivity (Fig. S4). Still, there is recent evidence on both individual and synergistic effects of climatic factors on plant community structure, species abundance, and succession. However, these aspects are not directly in the scope of this study.

### 4.3 Role of soil moisture in the CO_2_ fluxes

**Fig 7:**
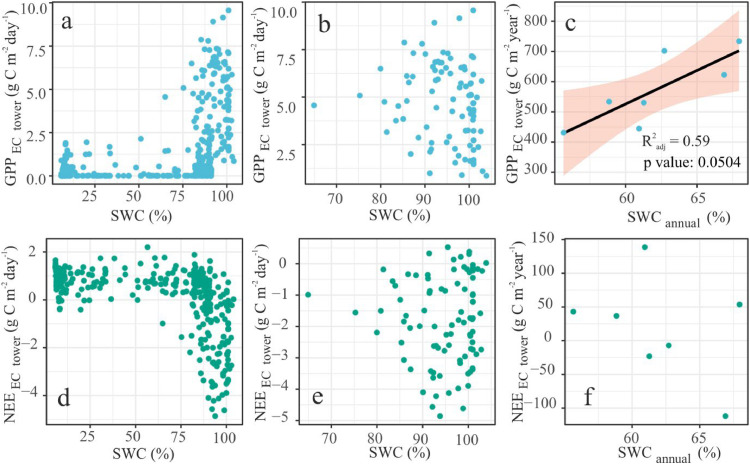
SWC does not explain variations in NEE and GPP. a: at weekly time-step for all seasons, b: growing season, & c: mean annual scale plotted against SWC. d, e, and f depict the relationship of NEE with SWC in the same temporal order as GPP.

It is worthwhile to note at this point that DALEC2 does not include soil moisture input or parameterisation of moisture-related abiotic factors. Soil moisture did explain almost 60% of the variations in mean annual estimates of GPP (Fig. 7c; R^2^_adj_ = 0.59, F-statistic: 9.273 on 1 and 5 DF, p-value: 0.05) as a positive linear trend similar to Evans et al. (2021). The mean annual estimates of SWC, filling around 60% to 70% of soil pore space, did not represent the saturated conditions of growing seasons as they were skewed from the winter-frozen and thus dry readings. Neither did weekly estimates of SWC explain any variation in the CO_2_ fluxes (Fig. 7a; & d). On closer inspection of the growing-season variability of C fluxes, we got a similar outcome (Fig. 7b & 7e). Apparently, all growing season variation in SWC (Fig. 7a & 7b) happens during saturated conditions. In the same vein, Laine et al. (2019) showed that water table did not have any impact on primary production in a sub-arctic Finnish fen. But these estimates might not be a realistic representation of the site conditions since it was flooded. Analogous outcomes were reported for leaf production in a bog and poor fen in the Southern Finland (Köster et al., 2023). Contrasting results were obtained for a Finnish rich fen (Köster et al., 2023). The site is reported to have contribution to GPP from algal production during the periods of inundation (DeColibus et al., 2017; Wyatt et al., 2012), especially in previously drier sites as opposed to sites with constant inundation (Kane et al., 2021). Hence it is likely that GPP was influenced by algal production during the flood years of 2014, 2016, 2017 and 2018. This might have contributed to underestimation of CARDAMOM simulated GPP compared to night-time partition EC data (Fig S1 & S2). In this study, we excluded soil moisture parameterisation to avoid adding to model redundancy.

### 4.4 Allocation to foliage is favoured over the fine root C pool

The greening trend seen in our findings also meant the foliage allocation is favoured over fine root and structural C pools. We think this is due to the climate warming and CO_2_ fertilisation effect at the site scale. This goes against the consensus that these effects force the plants to allocate more C to fine roots (Malhotra et al., 2020). But since the fen peatland was flooded for most of the study period, it did not have a necessity to retrieve water from the deeper soil horizon and hence the longer fine roots were not present. This could explain the relatively lower photosynthate allocation to fine roots (Weltzin et al., 2000). Similar fine root allocation patterns have been reported from the northern bog and fen ecosystems (Mäkiranta et al., 2018; Murphy and Moore, 2010). Several field manipulation experiments have portrayed the allocation patterns under warmer and wetter conditions. The experiments done by Weltzin et al. (2000) revealed a belowground allocation preference in both fen and bog systems under drier conditions and with higher water table levels aboveground allocation prevailed. This supports our findings (BNPP of 62.5 ± 7 gC m^-2^ year^-1^ against our 56.1 gC m^-2^ year^-1^ Figs. 2 & 5a). Their findings imply an increasing aboveground allocation under warming conditions which corroborate the foliage allocation preference seen in our investigation. It is unequivocally clear that in Bonanza Creek, flooding made the allocation dynamics complicated. We think this is probably why while CO_2_ fertilisation had an impact on the production, increasing temperature did not have the same efficacy. Laine et al. (2019) found that drying is the dominant factor in CO_2_ dynamics than warming, supporting the allocation patterns seen above.

### 4.5 Implications for the boreal zone peatlands

High latitudes, including the sub-Arctic, are experiencing unprecedented warming from the recent decades. This could lead to increased respiration of the CO_2_ trapped in the northern peatlands that cover 3.7 ± 0.5 million km^2^, shifting them from a sink of C to a source. On the other hand, the warming and rise in atmospheric CO_2_ concentration is enhancing C uptake during the growing season (IPCC, 2023). The fertilisation effect is to an extent offset by the increased peat respiration during the winter (Rafat et al., 2021). Peatlands with different trophic status and vegetation type will respond variously (Dieleman et al., 2015; Dorrepaal et al., 2004, 2009; Laine et al., 2019). Rich fens in particular may respond to climate change faster (Köster et al., 2023). Despite ongoing solid research on peatland C cycling and its climatic forcing, and better constraints on the uncertainty characterisation of C dynamics as seen in this study, the estimates are still largely uncertain (This study, Spawn et al., 2020). This demands the strengthening of peatland plant C specific inventories and model restructuration to suit peatland hydrology and ecosystems.

## Data availability

Eddy covariance data used in this study are openly available through Ameriflux: https://ameriflux.lbl.gov/ at https://doi.org/10.17190/AMF/1756433. The atmospheric CO_2_ concentration data which was used to substitute for the data gaps are available from https://doi.org/10.17190/AMF/1756434. The COPERNICUS LAI (300m) dataset is available through https://land.copernicus.eu/global/products/lai. Field data used in this study are published in https://doi.org/10.1139/cjfr-2014-0100, https://doi.org/10.1111/gcb.12041, and https://doi.org/10.1088/1748-9326/8/4/045029. inputs, outputs, and the codes used to generate the figures and supporting analysis are available through figshare: https://figshare.com at https://doi.org/10.6084/m9.figshare.25497736.v1. CARDAMOM and DALEC source codes are available to download from a GitHub repository: https://github.com/GCEL/, with registration provided upon request to T Luke Smallman.

## CRediT authorship contribution statement

**Sandeep Thayamkottu:** Writing – review & editing, Writing – original draft, Visualization, Validation, Project administration, Methodology, Investigation, Funding acquisition, Formal analysis, Data curation, Conceptualization. **T. Luke Smallman:** Writing – review & editing, Visualization, Validation, Supervision, Software, Resources, Methodology, Funding acquisition, Conceptualization. **Jaan Pärn:** Writing – review & editing, Visualization, Validation, Supervision, Resources, Methodology, Funding acquisition. **Ülo Mander:** Writing – review & editing, Visualization, Validation, Supervision, Resources, Methodology, Funding acquisition. **Eugénie S Euskirchen:** Writing – review & editing, Visualization, Validation, Resources, Methodology, Funding acquisition, Data curation. **Evan S Kane:** Writing – review & editing, Visualization, Validation, Resources, Methodology, Funding acquisition, Data curation.

## Declaration of competing interest

The authors declare that they have no known competing financial interests or personal relationships that could have appeared to influence the work reported in this paper.

## Data Availability

We have shared the DOI to the data used in the manuscript.

## Appendix Supplementary materials

Image, application 1
