# Supplementary material for: Greening of a boreal rich fen driven by CO2 fertilisation
Source: Agric For Meteorol. 2024 Dec 15;359:110261. doi: 10.1016/j.agrformet.2024.110261 (PMC11584299; doi:10.1016/j.agrformet.2024.110261)
Supplement: Supplementary file 1 [file mmc1.docx]

**Supplementary file for “Greening of a boreal rich fen driven by CO_2_ fertilisation”**

## SI Methods

We conducted exhaustive meta-analysis on the CO_2_ dynamics (focusing on internal C traits) of northern peatlands and other high latitude ecosystems including Arctic tundra. Key findings are summarised in SI table 3. These insights were used to update the model priors on fine root turnover rate, fine root C stock, and aboveground C stock (Table S1) and finally used a as a measure of validation in addition to the validation with EC tower data (Fig S2). The rest of the uniform parameter prior ranges used in the model were left as default (Bloom et al., 2016; Bloom & Williams, 2015). Additionally, we used a R_a_:GPP (1 – plant CUE) initial prior estimate of 0.62 ± 0.04 (Collalti et al., 2020; Hermle et al., 2010) and an initial prior for canopy efficiency of 16.9 gC m^-2^ day^-1^ with an SE of 7.5 gC m^-2^ day^-1^ (Kattge et al., 2011).

| **No.** | **Description** | **Prior range** | **Units** |
| --- | --- | --- | --- |
| P(1) | Decomposition of litter to SOM | 0.00001 - 0.01 | Fraction |
| P(2) | Autotrophic respiration fraction | 0.2-0.8 | Fraction |
| P(3) | Fraction of GPP allocated to foliage C pool | 0.01-0.5 | Fraction |
| P(4) | Fraction of GPP allocated to fine roots | 0.1-0.8 | Fraction |
| P(5) | Leaf lifespan | 1.001 - 6 | Years |
| P(6) | Woody C turnover rate | 0.000009 – 0.001 | Fraction / day |
| P(7) | Fine root C turnover rate | 0.0008 - 0.0004 | Fraction / day |
| P(8) | Litter C turnover rate | 0.0001141 – 0.02 | Fraction / day |
| P(9) | Soil organic C turnover rate | 10^-7^ - 10^-3^ | Fraction / day |
| P(10) | Temperature dependence exponent factor | 0.019 - 0.08 | - |
| P(11) | Photosynthetic canopy efficiency | 1.64 - 42 | gC m^-2^ day^-1^ |
| P(12) | Max bud burst day | 1 - 365.25 | Day of year |
| P(13) | Fraction of GPP allocated to labile | 0.01 -0.5 | Fraction |
| P(14) | Labile release period | 10 - 100 | days |
| P(15) | Max leaf fall day | 1 – 365.25 | days |
| P(16) | Leaf fall period | 20 - 150 | days |
| P(17) | Leaf C per area | 20 - 180 | gC m^-2^ |
| P(18) | Labile C pool at initial time | 1 – 2000 | gC m^-2^ |
| P(19) | Foliar C pool at time t | 1 – 2000 | gC m^-2^ |
| P(20) | Fine root C pool at time t | 1 – 750 | gC m^-2^ |
| P(21) | Above and below woody carbon pool at time t | 1 – 400 | gC m^-2^ |
| P(22) | Litter C pool at time t | 1 - 2000 | gC m^-2^ |
| P(23) | Soil organic C pool at time t | 200 - 125000 | gC m^-2^ |
| P(24) | Resilience factor for burned but not combusted C stocks | 0.1 – 1 | Fraction |
| P(25) | Combustion completeness factor for foliage | 0.01 – 0.99 | Fraction |
| P(26) | Combustion completeness factor for fine root and wood | 0.01 – 0.99 | Fraction |
| P(27) | Combustion completeness factor for soil | 0.001 – 0.1 | Fraction |
| P(28) | Combustion completeness factor for foliage and fine root litter | 0.01 – 0.99 | Fraction |

Table S1 Model parameters and their prior range.

| **Drivers** | **Unit** |
| --- | --- |
| DOY | Day of year |
| Minimum temperature | °C |
| Maximum temperature | °C |
| Mean temperature | °C |
| Incoming shortwave radiation | MJ m^-2^ day^-1^ |
| Atmospheric CO_2_ concentration | ppm |
| Precipitation | mm day^-1^ |

Table S2: Meteorological factors that drives DALEC2

## SI Results


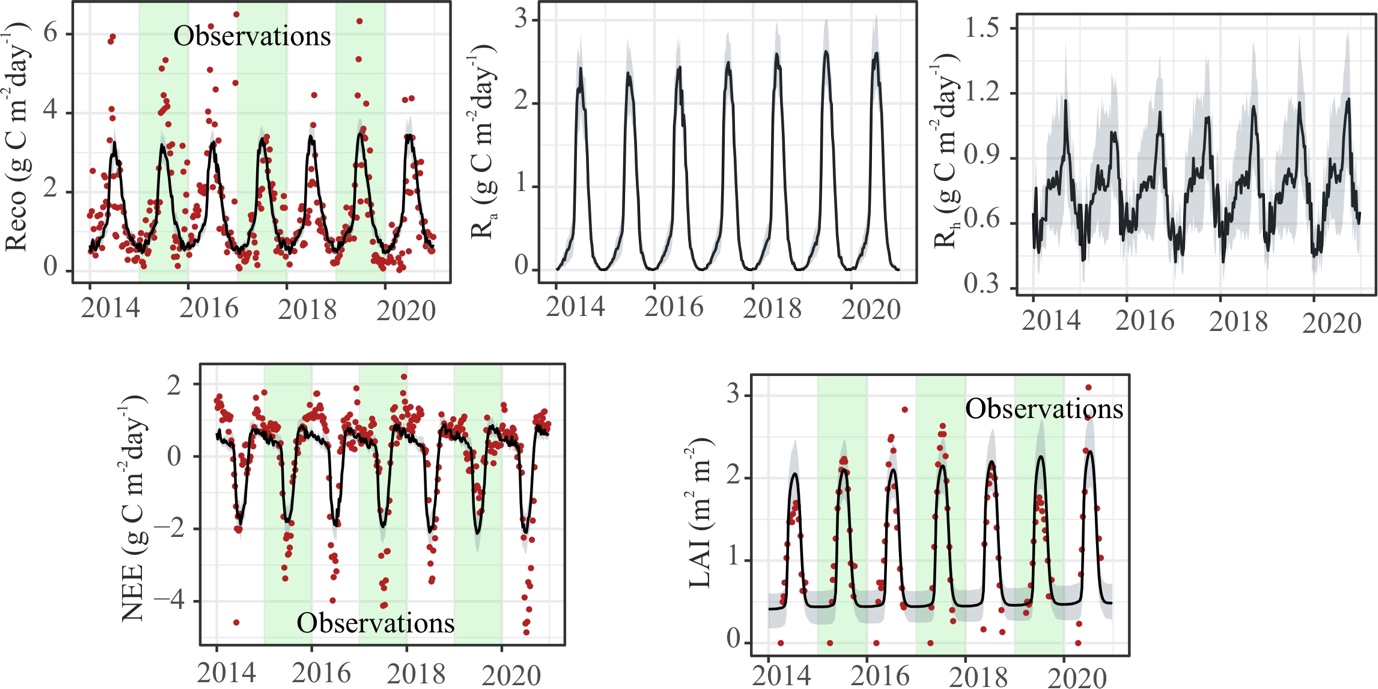


Figure S1: Time series of weekly timestep CO_2_ fluxes and LAI profiled by CARDAMOM for the period 2014-2020. The black line shows median estimates, and the shaded grey region shows the 95% CI. The points indicate EC tower observations. The observations from the shaded green region were not assimilated in CARDAMOM. These were used for validation (see Fig S2 below).


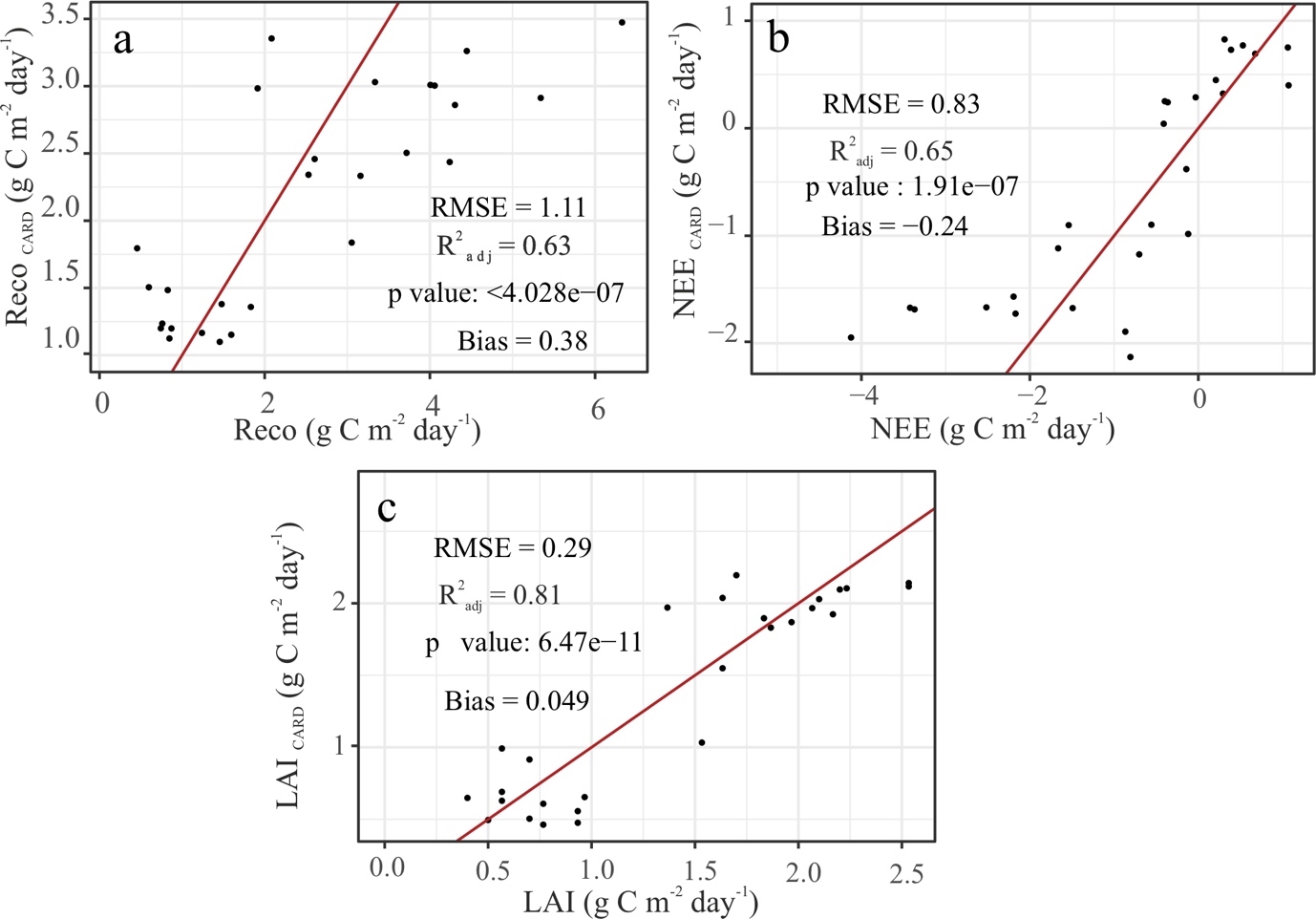


Figure S2: Weekly estimates of the CARDAMOM outputs validated against the data which were not part of the assimilation. The red colour represents the 1:1 line. Key statistics: RMSE, bias, R^2^_adj_, & p value are reported for each figure.

Calibrated DALEC outputs of NEE, Reco, GPP and LAI were validated using EC tower data (SI figure 2) which were not part of the assimilation (2015, 2017, and 2019). We used night-time partition GPP and Reco data for the validation. Since GPP were not assimilated, the whole 364 weekly-timestep data was used for validation.

| Site/region | Fine root carbon stock (<2 mm)  gC m^-2^ | Coarse root carbon stock (mostly 2-5 mm)  gC m^-2^ | Aboveground carbon stock  gC m^-2^ | | Total NPP  g C m^-2^ year^-1^ | | Aboveground NPP  gC m^-2^ year^-1^ | | Belowground NPP (fine root)  gC m^-2^ year^-1^ | | Turnover rate  (year ^-1^) | | Reference |
| --- | --- | --- | --- | --- | --- | --- | --- | --- | --- | --- | --- | --- | --- |
| Bonanza creek, Alaska^*^  (in-situ data) | 247.06 ± 140.86 |  | 282 ± 49.165 | | 214.33 ± 150.5 | | 186.5 | | 34.8 ± 10.165 | | 0.14 ± 0.072 | | (Churchill et al., 2015; McConnell et al., 2013) |
| CARDAMOM  (95% CI) | 252.8 (136.5 to 406.9) | 302.78 | AGC = 172.675  Of this AGC Foliage = 139.4 (93.1 to 173.9),  Stem C = 34.22 | | 289 (248.3 to 332.4) | | Woody (includes coarse root) + foliage = 86.2 (41.6 to 130.4)  16.5 (3.4 to 97.8) | | 54.7 (31.4 to 92.6) | | 0.22 | |  |
| Lompolojänkkä (Arctic), Finland | 134.41 (after 3 years in in-growth bag (0-60 cm). estimated from the original data) |  |  | |  | |  | |  | |  | | (Bhuiyan et al., 2023) |
| Lakkasuo (Southern Finnish sedge dominated Bog plot) | 121.15±20.6 |  |  | |  | |  | | 59.2±12.5 | | 0.48 | | (Lampela et al., 2023) |
| Mer Bleu, Canada (Bog) | 175±80 (Hummock)  295±275 (Lawn) | 825±405  (Hummock)  395±265  (Lawn) | Hummock: 500±330; Lawn:165±60  (mostly contributed by the Carex sedge species) | |  | |  | |  | |  | | (Murphy & Moore, 2010) |
| Germany (forested temperate peatland, species specific) | 25.85 ± 10.37 (<1mm) |  |  | |  | |  | |  | |  | | (Schwieger et al., 2021) |
| Disko island, Greenland | 142.5 ± 14 |  |  | |  | |  | | 42 ± 0.672 | | 0.29 ± 0.048 | | (D’Imperio et al., 2018) |
| Arctic tundra  (Multi-site) | 0.05-1850 (Total root carbon stock)  Median – 175  Sedge species – 0-500 |  | |  | |  | |  | | >0.2 | | (Iversen et al., 2015) | |
| Arctic tundra  (Wetland gradient) | 236 ± 39 | 269 ± 120 | Stem = 219 ± 68  Leaf = 52 ± 5 | |  | |  | |  | |  | | (Sloan et al., 2013) |
| Sedge dominated Arctic tundra.  (2002-2005) | 125 ± 25 |  |  | |  | |  | | 79.5 ± 33.5 (0-40 cm) (ambient)  72.5 ± 16 (warm growing season)  44 ± 14 (0-25 cm) | |  | | (Sullivan et al., 2008) |
| Sedge dominated Arctic peatland |  |  |  | |  | |  | | 59.5 ± 24 | |  | | (Sullivan & Welker, 2005) |
| Arctic tundra (multi-site meta-analysis) | 426.5±46.5 | 129.5 ± 25.5 | |  | |  | |  | |  | | (Wang et al., 2016) | |
| Halssiaapa, (Boreal fen, Northern Finland) |  | 145.75 ± 104.7 (Biomass harvest, July 2014)  118.15 ± 39.3 (Landscape level all EO based)  117.75 ± 38.45 (Landscape level Hyperspectral)  123.95 ± 35.65 (Landscape level Without hyperspectral) | |  | |  | |  | |  | | (Räsänen et al., 2019) | |
| Brøggerhalvøya (north-western Svalbard)  (Growing season moss NPP) |  |  | |  | | 2012: 205 gC m^-2^,  2013: 207,  2014: 143 | |  | |  | | (Nakatsubo et al., 2023) | |
| Alaska (Two wetlands) |  | Leaf: 21  Moss (visual interpretation from the figure): 50  Lichen (visual interpretation from the figure): 10 | |  | |  | |  | |  | | (Williams & Rastetter, 1999) | |

Table S3 : A comparison of CARDAMOM outputs against published literature on high latitude ecosystems

We followed Wang et al (2018) and partitioned CARDAMOM estimates of structural C to aboveground and coarse root carbon (SI table 3). Wang et al; (2018) reported that ~ 0.76 ± 0.02 of the total biomass belonged to belowground biomass and a 0.47 ± 0.08 of the total biomass was fine root biomass. This gives an estimate of ~ 302.78 gC m^-2^ as coarse root C and a 34.22 gC m^-2^ as aboveground structural C in the fen peatland ecosystem. This is a highly probable amount of C partition for a sedge dominated peatland.


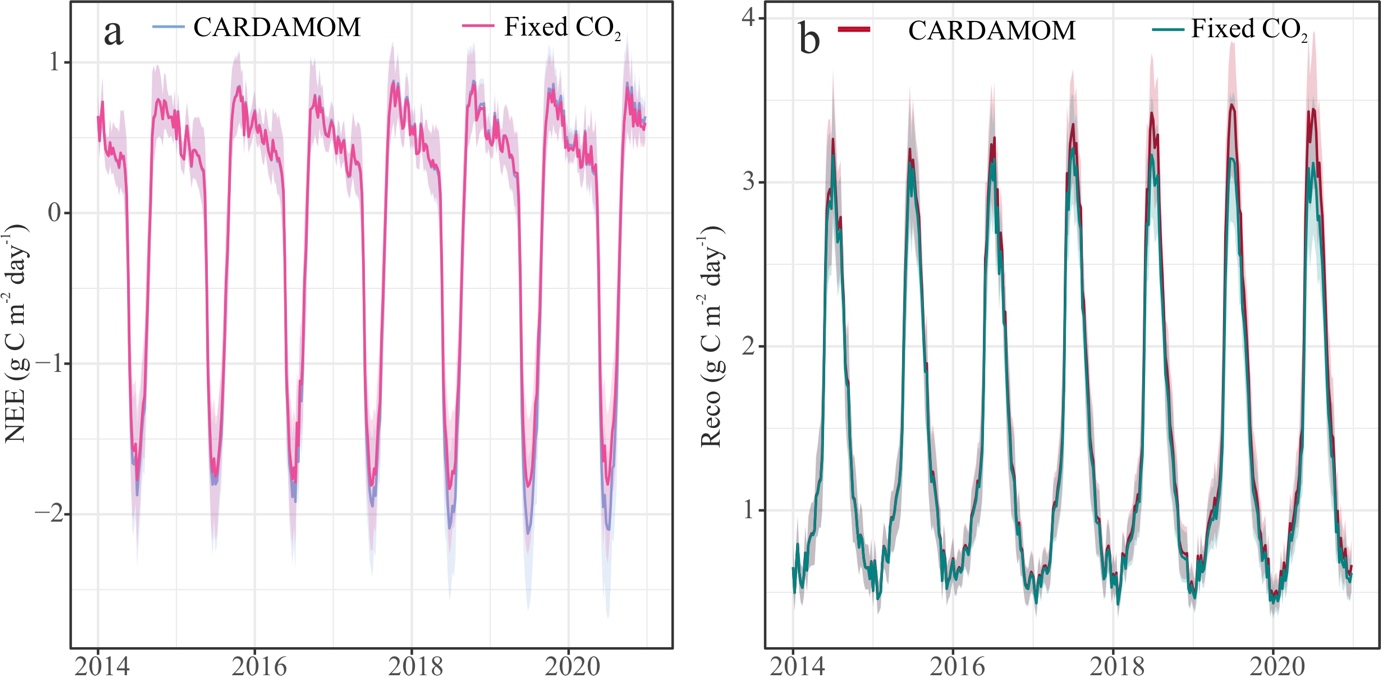


Figure S3: comparison between the weekly time series a: NEE, and b: Reco, profiled by CARDAMOM, and Experiment 1. (For more details on the synthetic experiments, refer the methods section) The shaded regions represent the respective 95% CI estimated by CARDAMOM.


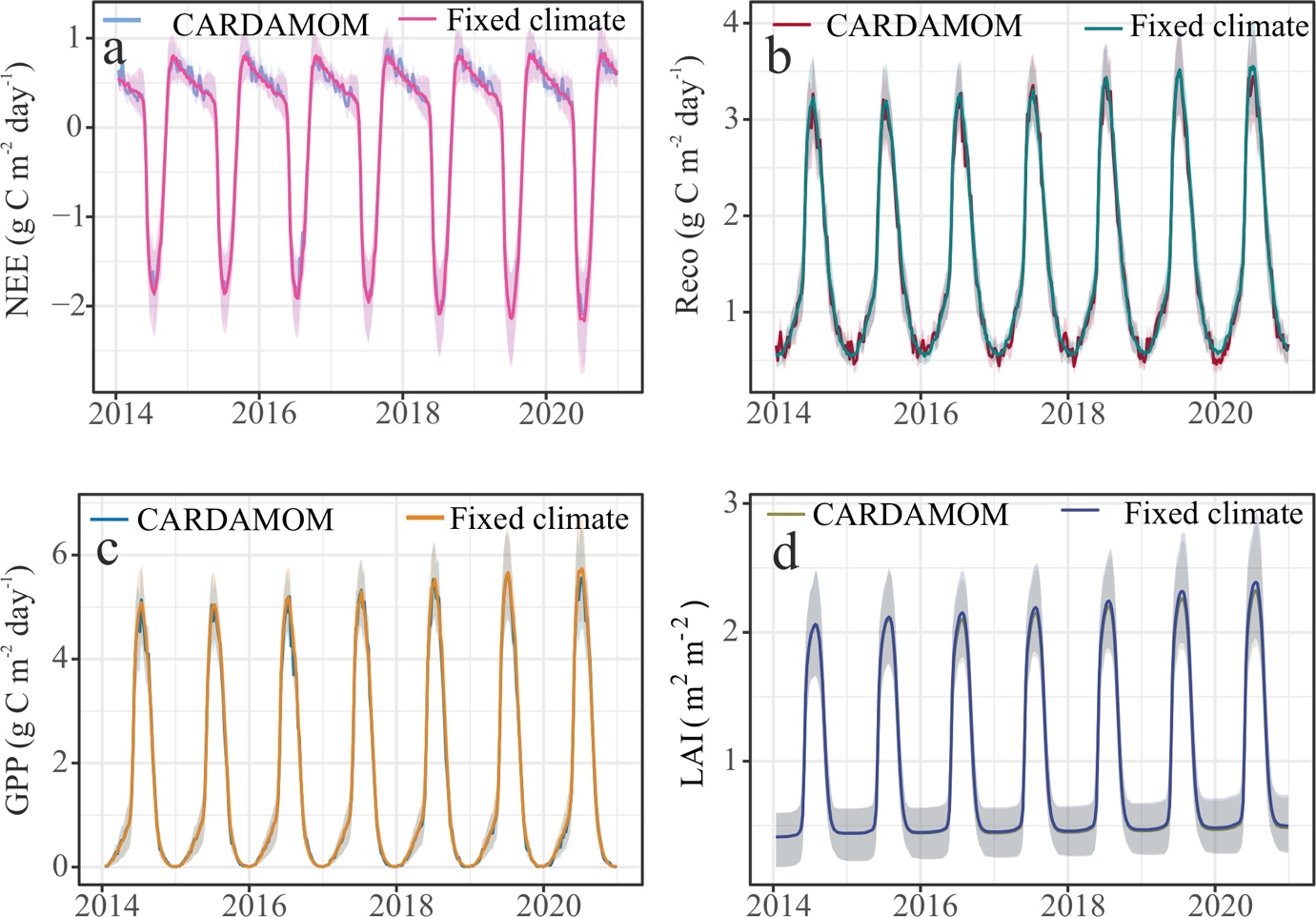


Figure S4: comparison between the weekly time series a: NEE, b; Reco; c: GPP, and d: LAI, profiled by CARDAMOM, and Experiment 2. (For more details on the synthetic experiments, refer the methods section) The shaded regions represent the respective 95% CI estimated by CARDAMOM.


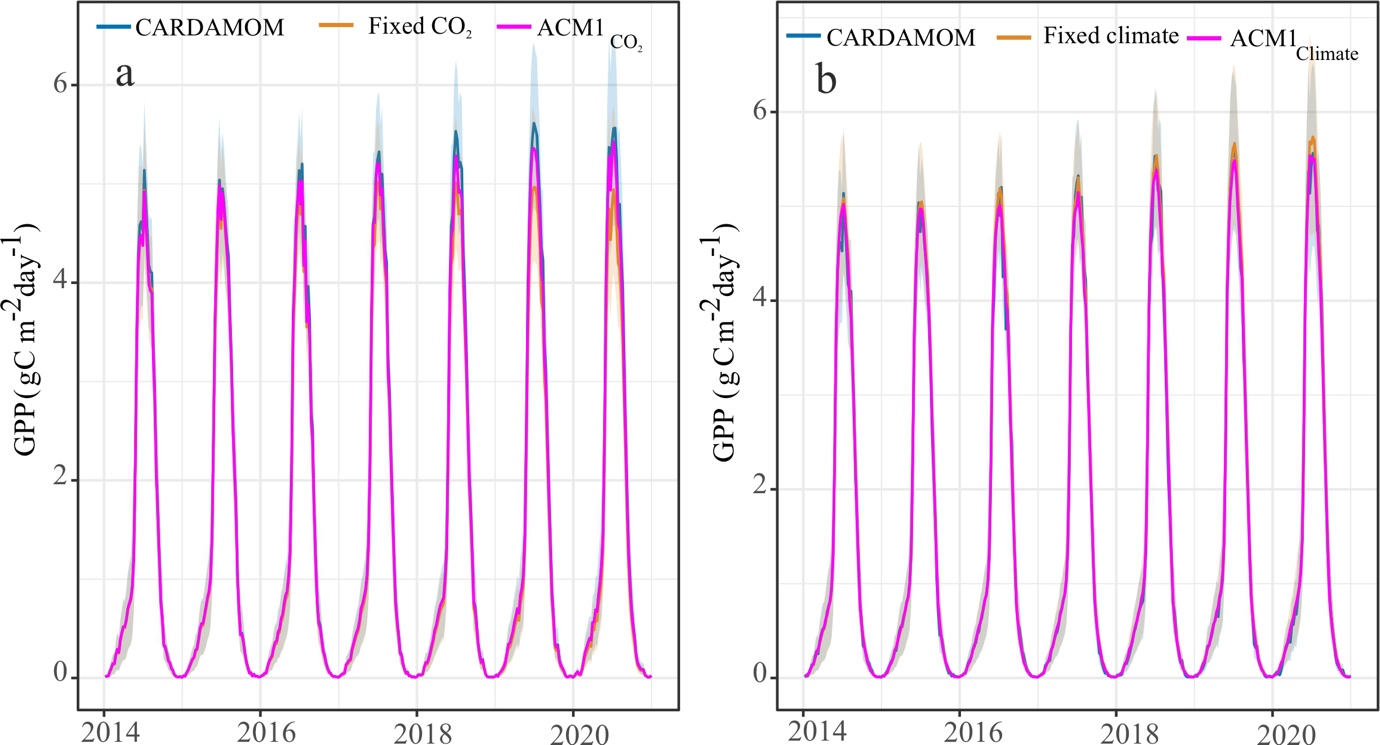


Figure S5: comparison between the weekly time series estimates of GPP simulated by a: CARDAMOM, Fixed CO_2_ experiment, and ACM1_CO2_ b; CARDAMOM, Fixed climate experiment, and ACM1_Climate_. ACM1_CO2_ and ACM1_Climate_ are GPP estimated by the ACM-1 model with fixed CO_2_ and fixed climate meteorology data from the Fixed CO_2_ experiment and Fixed climate experiment respectively. The shaded regions represent the respective 95% CI.

# SI References

Bhuiyan, R., Mäkiranta, P., Straková, P., Fritze, H., Minkkinen, K., Penttilä, T., Rajala, T., Tuittila, E.S. & Laiho, R. (2023). Fine-root biomass production and its contribution to organic matter accumulation in sedge fens under changing climate. *Science of The Total Environment*, *858*, 159683. https://doi.org/10.1016/j.scitotenv.2022.159683

Bloom, A. A., Exbrayat, J. F., Van Der Velde, I. R., Feng, L., & Williams, M. (2016). The decadal state of the terrestrial carbon cycle: Global retrievals of terrestrial carbon allocation, pools, and residence times. *Proceedings of the National Academy of Sciences*, *113*(5), 1285–1290. https://doi.org/10.1073/pnas.1515160113

Bloom, A. A., & Williams, M. (2015). Constraining ecosystem carbon dynamics in a data-limited world: Integrating ecological "common sense"; in a model–data fusion framework. *Biogeosciences*, *12*(5), 1299–1315. https://doi.org/10.5194/bg-12-1299-2015

Churchill, A. C., Turetsky, M. R., McGuire, A. D., & Hollingsworth, T. N. (2015). Response of plant community structure and primary productivity to experimental drought and flooding in an Alaskan fen. *Canadian Journal of Forest Research*, *45*(2), 185–193. https://doi.org/10.1139/cjfr-2014-0100

Collalti, A., Ibrom, A., Stockmarr, A., Cescatti, A., Alkama, R., Fernández-Martínez, M., Matteucci, G., Sitch, S., Friedlingstein, P., Ciais, P., Goll, D. S., Nabel, J. E. M. S., Pongratz, J., Arneth, A., Haverd, V., & Prentice, I. C. (2020). Forest production efficiency increases with growth temperature. *Nature Communications*, *11*(1), 5322. https://doi.org/10.1038/s41467-020-19187-w

D’Imperio, L., Arndal, M. F., Nielsen, C. S., Elberling, B., & Schmidt, I. K. (2018). Fast responses of root dynamics to increased snow deposition and summer air temperature in an arctic wetland. *Frontiers in Plant Science*, *9*, 1258. https://doi.org/10.3389/fpls.2018.01258

Hermle, S., Lavigne, M. B., Bernier, P. Y., Bergeron, O., & Paré, D. (2010). Component respiration, ecosystem respiration and net primary production of a mature black spruce forest in northern Quebec. *Tree Physiology*, *30*(4), 527–540. https://doi.org/10.1093/treephys/tpq002

Iversen, C. M., Sloan, V. L., Sullivan, P. F., Euskirchen, E. S., McGuire, A. D., Norby, R. J., Walker, A. P., Warren, J. M., & Wullschleger, S. D. (2015). The unseen iceberg: Plant roots in arctic tundra. *New Phytologist*, *205*(1), 34–58. https://doi.org/10.1111/nph.13003

Kattge, J., Díaz, S., Lavorel, S., Prentice, I. C., Leadley, P., Bönisch, G., Garnier, E., Westoby, M., Reich, P. B., Wright, I. J., Cornelissen, J. H. C., Violle, C., Harrison, S. P., Van BODEGOM, P. M., Reichstein, M., Enquist, B. J., Soudzilovskaia, N. A., Ackerly, D. D., Anand, M., … Wirth, C. (2011). TRY – a global database of plant traits. *Global Change Biology*, *17*(9), 2905–2935. https://doi.org/10.1111/j.1365-2486.2011.02451.x

Lampela, M., Minkkinen, K., Straková, P., Bhuiyan, R., He, W., Mäkiranta, P., ... & Laiho, R. (2023). Responses of fine-root biomass and production to drying depend on wetness and site nutrient regime in boreal forested peatland. *Frontiers in Forests and Global Change*, *6*, 1190893. https://doi.org/10.3389/ffgc.2023.1190893

McConnell, N. A., Turetsky, M. R., McGuire, A. D., Kane, E. S., Waldrop, M. P., & Harden, J. W. (2013). Controls on ecosystem and root respiration across a permafrost and wetland gradient in interior Alaska. *Environmental Research Letters*, *8*(4), 045029. https://doi.org/10.1088/1748-9326/8/4/045029

Murphy, M. T., & Moore, T. R. (2010). Linking root production to aboveground plant characteristics and water table in a temperate bog. *Plant and Soil*, *336*(1), 219–231. https://doi.org/10.1007/s11104-010-0468-1

Nakatsubo, T., Hirota, M., Kishimoto-Mo, A. W., Oura, N., & Uchida, M. (2023). Carbon exchange and primary production in a High-Arctic peatland in Svalbard. *Polar Research*, *42*. https://doi.org/10.33265/polar.v42.8541

Räsänen, A., Juutinen, S., Aurela, M., & Virtanen, T. (2019). Predicting aboveground biomass in Arctic landscapes using very high spatial resolution satellite imagery and field sampling. *International Journal of Remote Sensing*, *40*(3), 1175–1199. https://doi.org/10.1080/01431161.2018.1524176

Schwieger, S., Kreyling, J., Couwenberg, J., Smiljanić, M., Weigel, R., Wilmking, M., & Blume-Werry, G. (2021). Wetter is better: Rewetting of minerotrophic peatlands increases plant production and moves them towards carbon sinks in a dry year. *Ecosystems*, *24*(5), 1093-1109. https://doi.org/10.1007/s10021-020-00570-z

Sloan, V. L., Fletcher, B. J., Press, M. C., Williams, M., & Phoenix, G. K. (2013). Leaf and fine root carbon stocks and turnover are coupled across Arctic ecosystems. *Global Change Biology*, *19*(12), 3668–3676. https://doi.org/10.1111/gcb.12322

Sullivan, P. F., Arens, S. J., Chimner, R. A., & Welker, J. M. (2008). Temperature and microtopography interact to control carbon cycling in a high arctic fen. *Ecosystems*, *11*, 61-76. https://doi.org/10.1007/s10021-007-9107-y

Sullivan, P. F., & Welker, J. M. (2005). Warming chambers stimulate early season growth of an arctic sedge: Results of a minirhizotron field study. *Oecologia*, *142*(4), 616–626. https://doi.org/10.1007/s00442-004-1764-3

Wang, P., Heijmans, M. M., Mommer, L., van Ruijven, J., Maximov, T. C., & Berendse, F. (2016). Belowground plant biomass allocation in tundra ecosystems and its relationship with temperature. *Environmental Research Letters*, *11*(5), 055003. https://doi.org/10.1088/1748-9326/11/5/055003

Williams, M., & Rastetter, E. B. (1999). Vegetation characteristics and primary productivity along an arctic transect: Implications for scaling-up. *Journal of Ecology*, *87*(5), 885–898. <https://doi.org/10.1046/j.1365-2745.1999.00404.x>
